# Food Allergy and Foodservice: A Comparative Study of Allergic and Non-Allergic Consumers’ Behaviors, Attitudes, and Risk Perceptions

**DOI:** 10.3390/nu17182916

**Published:** 2025-09-09

**Authors:** Fatemeh Shirani, Silvia Dominguez, Jérémie Théolier, Jennifer Gerdts, Kate Reid, Sébastien La Vieille, Samuel Godefroy

**Affiliations:** 1 Food Risk Analysis and Regulatory Excellence Platform (PARERA), Institute of Nutrition and Functional Foods, Department of Food Science, Université Laval, Quebec, QC G1V 0A6, Canada; silvia.dominguez.1@ulaval.ca (S.D.); jeremie.theolier@fsaa.ulaval.ca (J.T.); sebastien.lavieille@hc-sc.gc.ca (S.L.V.); samuel.godefroy@fsaa.ulaval.ca (S.G.); 2 Food Allergy Canada, 505 Consumers Drive, Suite 507, Toronto, ON M2J 4A2, Canada; jgerdts@foodallergycanada.ca (J.G.); kreid@foodallergycanada.ca (K.R.); 3 Health Canada, Food Directorate, 251 Sir Frederick Banting Driveway, Ottawa, ON K1A 0K9, Canada

**Keywords:** food allergy, foodservice establishments, restaurants, consumer survey, convenience sample

## Abstract

**Background:** Food-allergic reactions in restaurants often result from miscommunication between customers with allergies and staff, or from staff members’ insufficient knowledge of food allergies. This study examined the behaviors, attitudes, and risk perceptions of food-allergic consumers when dining out or ordering from foodservice establishments (FSEs) compared to consumers without food allergies. **Methods:** A representative pan-Canadian survey was conducted amongst three groups: one of individuals without food allergies (n = 500) and two of food-allergic individuals (allergic-convenience sample [n = 500] and allergic-general population [n = 500]). The convenience sample comprised members of Food Allergy Canada, a national patient advocacy organization. Some participants with food allergies had experienced reactions linked to an FSE (43% convenience, 27% general). Weighted responses from food-allergic groups were compared to those of non-allergic ones using chi-square (*p* < 0.05). Statistical comparison between allergic groups was not attempted due to inherent differences in their allergic condition. **Results:** In several questions, responses from the non-allergic group differed significantly from those of the allergic-convenience sample, but not from those of the allergic-general population. Food-allergic-convenience respondents were more likely to avoid ordering food or dining out than non-allergic ones, with the highest avoidance (66%) noted for third-party platforms. Cost was the main barrier for non-allergic and allergic-general populations, whereas the allergic-convenience sample prioritized allergy-related concerns. Although at a lower rate than for participants with food allergies, food allergies influenced restaurant selection for 44% of participants without food allergies when dining with individuals outside their household. Most allergic respondents perceived that FSEs underestimate the seriousness of food allergies (82% convenience, 71% general), yet they felt safe while dining out (60% convenience, 85% general), pointing at loyalty to specific FSEs as a risk mitigation strategy. **Conclusions:** This study highlights a potentially higher burden of disease (psychological and social strain, reduced quality of life) among a subgroup of the food-allergic population (convenience sample), as reflected in their behaviors, attitudes, and risk perceptions towards meals prepared in FSEs. Nevertheless, both allergic groups expressed shared concerns and needs related to safety (e.g., ingredient disclosure for all menu items, prevention of allergen cross-contact, ability of an FSE to offer a safe meal, establishing clear communication processes for allergy-related information), which FSEs and regulators should consider when designing risk management strategies.

## 1. Introduction

Food allergies are life-threatening conditions affecting approximately 6 to 13% of the population worldwide [1]. In Canada, the prevalence of food allergy based on history and/or physician diagnosis is estimated to be at approximately 6% [2,3]. Despite advances in immunotherapy treatments [4,5,6], strict avoidance of the identified culprit allergen(s) remains the primary strategy to prevent allergic reactions [1,7,8].

Most food-allergic reactions occur at home, followed by those occurring at restaurants and schools [9,10]. Overall, 21–31% of food-allergic reactions are estimated to occur in restaurants [11]. In the United States, between 2017 and 2019, 31% of adult and 13% of pediatric allergic reactions to food were estimated to occur while dining out [9]. Also, 16.8% of participants of a survey related to allergic reactions in the COVID-19 era indicated suffering allergic reactions from restaurant take-out food [12]. Reportedly, a considerable number of food-induced allergic reactions that occur while dining away from home are due to a lack of clear communication about food allergy risks [13]. Also, most fatal cases of food anaphylaxis occur when allergic individuals consume their food in another country where language barriers and unfamiliar cuisine can contribute to the risk [14]. Consequently, while dining out offers social benefits, it may pose health risks for individuals with food allergies [15,16] due to potential inadvertent exposure to allergens [17]. Previous research highlights the significance of effectively communicating food allergies as a crucial risk management tool and acknowledges the challenges allergic consumers face when consuming food prepared in FSEs [18,19]. However, limited attention has been paid to the nature of allergy risk-related conversations between consumers and restaurant staff [20,21]. Consumer behaviors, needs, perceptions, and expectations are subject to quick evolution, which makes it even more important to be studied [22]. Unfortunately, there is limited research on allergic consumers’ knowledge, attitudes, and behaviors related to their dining out experiences [21,23,24]. Kwon et al. (2020) conducted a survey of restaurant customers with food allergies in the United States and found that most had high allergy knowledge scores, were able to recognize typical causes of food allergy reactions in FSEs, preferred chain restaurants, and applied a combination of strategies to select a restaurant or meal [23]. In another survey, López-Calvo et al. (2022) noted that food-allergic consumers in Costa Rica expect allergy declaration in FSE menu items and for all food handlers to undergo food allergy training [24]. As a result of limited data, restaurateurs’ lack of understanding of these factors may impede their ability to implement effective strategies for preventing allergic reactions in FSEs [23]. A better understanding of food-allergic (FA) consumers’ challenges when dining out could also inform the development of enhanced allergen management and ingredient declaration policies targeting FSEs [13,21] and thus increase safe food choices for allergic consumers [17]. These policies have been implemented in several jurisdictions, although specific requirements and enforcement vary [21]. Specifically, in Europe, it is mandated by law that providers of non-prepacked foods must supply clear information regarding the presence of allergens in their products, with the aim of enhancing consumer awareness and safety [17,19,25]. The UK also adheres to this requirement under the Food Information to Consumers regulation (previously part of EU law), with enhanced details on the manner of declaration [26]. Similar regulations have also been proposed in Saudi Arabia [27] and Australia/New Zealand [17]. In Canada, only prepackaged foods are required to follow allergen management provisions [28], which would only concern FSEs if they sell prepackaged foods that were prepared and packed at off-site premises.

Indeed, FSEs lag behind the prepackaged food industry in implementing allergen management systems, mainly due to operational challenges, including limited space, shared equipment, fluctuating menus, and high employee turnover [17], as well as the absence of national or provincial regulations [21,29]. In addition, the multiple and fast-paced steps involved in the ordering, preparation, and serving/delivery of a meal at an FSE may lead to handling (e.g., cross-contact) and/or communication (e.g., between front-of-house and back-of-house staff) errors, which could result in unintentional allergen presence [9,21,23,30,31,32]. Furthermore, food allergies are often not included in FSEs’ staff training programs [33]. Nevertheless, these legitimate challenges “should not be used as a justification for continued inaction” [34] regarding allergen management regulations and transparency in FSEs. For example, enhanced mandatory requirements for food allergy training in FSEs would contribute to the harmonization of their working processes [31]. Moreover, the mandatory declaration of allergens for food sold at FSEs, if required, must be accompanied by the implementation of effective allergen management practices in these FSEs [34], including cross-contact prevention measures and regular staff training aiming to improve communication related to food allergens [23], as well as vendor assurance systems and (bulk) food label requirements [17]. In fact, to prevent allergic reactions due to food prepared in FSEs, a collaborative effort between foodservice operators, regulators, food suppliers, and FA customers is fundamental [21,29]. In this context, the availability of thorough information regarding food allergen ingredients combined with consumers’ disclosure of allergen restrictions to well-informed FSE staff is regarded as an efficient risk management strategy [9,13]. Additionally, individuals with food allergies should always carry an epinephrine device while dining out and avoid allergens to which they react [35,36].

Data on allergic consumers’ approaches to risk communication when dining out or ordering meals prepared in FSEs are needed to identify challenges and propose avenues for improvement. Surveys are a well-established methodology to obtain consumer data; for example, on food safety in general [37,38,39], including studies on food allergies [40]. When targeting specific sub-populations, consumer studies often rely on convenience sampling rather than random selection [41]. A convenience sample is a non-probability sample where units are selected based on availability and convenience [40,42,43]. This sampling approach is inexpensive and quick [40,43] and is used in different research areas. However, the potential for biased results associated with convenience sampling [44] must be considered when designing the study and interpreting its results.

Thus, the aim of this study was to document Canadian FA consumers’ behaviors, attitudes, and risk perceptions while dining out or ordering meals from FSEs and to establish if they differ from those of non-allergic consumers. In addition, two different Canadian FA groups were considered in this survey, and their responses were compared to those of participants without food allergies, to explore the potential implications of recruiting allergic subjects from a convenience sample and from the general population. The results of this study may inform enhanced policies on allergen management and declaration in FSEs and facilitate the identification of strategies to improve this population’s experience and safety when dining out or ordering meals from FSEs.

## 2. Materials and Methods

### 2.1. Survey Instrument

An online questionnaire (Supplementary Material) was designed by Food Allergy Canada (a food-allergic consumer association) to examine and compare the behaviors, attitudes, and risk perceptions of food-allergic and non-allergic consumers while dining out or ordering meals from FSEs. The survey consisted of 33 questions (Supplementary Material) divided into three sections: (1) screening and profiling (e.g., place of residence, age, allergy characteristics); (2) foodservice behaviors, attitudes, and risk perceptions (e.g., dining/ordering frequency, spending habits, barriers, risk perceptions, incidents, safety concerns, loyalty); and (3) basic data (e.g., gender, income). The questions were designed using Likert scales, multiple-choice, and open-ended formats. The survey was issued in both French and English, with translations verified for accuracy. The survey was programmed and executed using the Decipher (Forsta) platform.

### 2.2. Target Population

Three groups of respondents were considered: (1) food-allergic individual members of Food Allergy Canada’s network (n = 500), referred to as the “FA-convenience sample”; (2) food-allergic individuals from the general population recruited through an external panel (n = 500), referred to as the “FA-general population”; and (3) individuals without food allergies recruited through an external panel (n = 500), referred to as the “non-FA sample”.

For groups sourced through an external panel, a representative sample of adults residing exclusively in British Columbia, the Canadian Prairies (i.e., Alberta, Saskatchewan, Manitoba), Ontario, Quebec, or Atlantic Canada (i.e., New Brunswick, Newfoundland and Labrador, Nova Scotia, Prince Edward Island) was obtained. Details on how sample representativity was obtained (target number of complete responses per province and assignment of weights) are provided in the Supplementary Materials. Participants had to be 18 years or older, and those with food allergies were required to either have a food allergy themselves or have a child with a food allergy that had been diagnosed or confirmed by a medical professional.

### 2.3. Data Collection

Food Allergy Canada distributed the online survey via email to their members (allergic-convenience sample). A 3rd-party service provider (Supplementary Material) distributed the online survey via email to the general population groups (allergic-general population and non-allergic). The survey was open between October and November 2023. Participants were informed of the study’s purpose, and blanket consent was obtained before starting the survey. Participation was voluntary, and anonymized answers were collected and shared for statistical analysis. The Multi-faculty Research Ethics Committee of Université Laval (dossier 2024-136) exempted the survey from full board review, as only secondary use of anonymous data was considered.

### 2.4. Data Analysis

To account for sociodemographic differences among respondents and thus allow for comparison with the non-FA sample, responses from each group of FA participants (FA-convenience sample and FA-general population) were weighted considering region, gender, age, and household income. Statistical analysis was based on weighted sample sizes (Table 1). For multiple-choice and Likert scale questions, differences between proportions of each group of FA (FA-convenience sample and FA-general population) and non-FA participants’ responses were compared using chi-square. Significant differences were identified considering *p*-value < 0.05. Responses to open-ended questions (Question 20b—Supplementary Material) were manually coded by the 3rd-party service provider based on the identified themes. Responses were first reviewed in full for familiarization with the subject. Next, a senior coder developed a code frame using a subset of verbatims. The code frame was developed inductively based on the identification of recurrent ideas, themes, and patterns. Then, the code was applied to the full set of collected verbatims, with additions/modifications made to the code list as warranted through the coding process.

All statistical analyses were conducted in R Studio [45], R version 4.4.1, and figures were produced using the ggplot2 [46,47] and Likert [48] packages. Comparison between FA groups (FA-convenience sample and FA-general population) was not attempted due to the inherent differences between these 2 populations (Table 1).

## 3. Results

### 3.1. Margin of Error

The survey respondents included 1000 FA individuals and 500 non-FA individuals living in Canada. For individuals without food allergies (38,977,380), considering a total population of 41,465,298 in Canada [49], the sample size (500) provides a 4% margin of error at a 95% confidence level. For allergic individuals, considering a prevalence of food allergy (based on history and/or physician diagnosis) roughly at 6% of the Canadian population [2,3], the sample size (1000) provides a 3% margin of error at a 95% confidence level. This means that our results are within 4% and 3 % of the real non-allergic and allergic population value, respectively, 95% of the time.

### 3.2. Respondents’ Characteristics

A summary of the sociodemographic characteristics of the respondents from the three groups studied, as well as the allergy characteristics of the FA respondents, is presented in Table 1. Intrinsic differences between the two groups of FA individuals, FA-convenience sample (i.e., members of Food Allergy Canada’s network), and FA-general population (i.e., recruited through an external panel), regarding the nature of their food allergy (e.g., diagnosing specialist, number of years since diagnosis, number of allergies, prevalence of most common allergen, prevalence of epinephrine prescription), were noted.

### 3.3. Frequency of Consumption of Food Prepared in Foodservice Establishments (FSEs)

Frequency of consumption of food prepared in FSEs, procured through three different formats (i.e., dining in a restaurant, ordering take-out or delivery from a restaurant, ordering delivery through a third-party service such as Skip and Uber Eats), varied per group of respondents (i.e., non-FA sample, FA-convenience sample, and FA-general population) (Table 2).

As shown in Table 2, most respondents from each group dined at a restaurant, or ordered take-out or delivery directly from a restaurant, less than once per week. An exception was noted for the FA-convenience sample, whose most common frequency for ordering through a third-party was “never”. Furthermore, these respondents were more likely (*p* < 0.001) to avoid dining out and ordering food (both formats) than non-allergic ones. However, 9% of the FA-convenience sample, 1% of the allergic-general sample, and 2% of the non-allergic sample indicated never consuming meals prepared in FSEs (Supplementary Material Question 10).

Respondents who reported consuming food prepared in an FSE were also questioned about their choice of establishment type (i.e., fast food/quick service, full-service chains, independent, specialized in a specific type of international cuisine) (Table 3).

As shown in Table 3, the most frequent consumption rate reported by all three groups of respondents for all four types of FSEs studied was “less than once per week”. For all respondents, the highest consumption rates were reported for fast food/quick service establishments. No significant differences were noted between the frequencies of consumption of the non-FA sample and FA-general population for any of the four types of FSEs studied. The FA-convenience sample was more (*p* < 0.001) likely to avoid fast food/quick service establishments, independent restaurants, and restaurants serving a specific type of international cuisine than non-allergic respondents. This avoidance was more pronounced for FSEs serving a specific type of international cuisine than for the other three types of FSEs studied. These respondents were also less likely (*p* < 0.05) to report high consumption frequencies than non-allergic ones.

### 3.4. Barriers When Dining Out/Ordering Food

Survey participants were also asked to identify barriers when it comes to dining out/ordering food from an FSE. Apart from common factors (e.g., related to cost or convenience) presented to all groups of respondents, FA respondents were presented with additional answer choices (i.e., allergy-related). The most common barriers for each group are presented in Table 4.

As shown in Table 4, cost (an option presented to all respondents) was the most frequent barrier when it comes to dining out/ordering food from an FSE for the non-FA sample and FA-general population. The FA-convenience sample was less prone to consider cost as a barrier (13th choice = 28% when dining out and 11th choice = 25% when ordering a meal). In addition, after cost, non-FA respondents were mainly concerned about convenience and health. The FA-convenience sample seemed more concerned with allergy-related matters. Apart from cost, similar barriers were shared by the FA-general population, although at a lower rate. Furthermore, most barriers were rated above 50% by the FA-convenience sample when dining out or ordering meals, in contrast to other participants (non-FA sample and FA-general population; Table 4).

### 3.5. Cost of Dining Out/Ordering Food

Regarding the amount typically spent when dining out (Supplementary Material Question 14a), all three groups reported between CAD 25 and 100 per person as the most frequent range (71% non-allergic, 74% allergic-convenience, 70% allergic-general). The FA-convenience sample (1%) were less likely (*p* < 0.05) to spend larger amounts (i.e., between CAD 101 and 200 per person) than non-FA respondents (4%) when dining out. The likelihood of the non-FA and FA-general population spending larger amounts when dining out was not significantly different.

When it comes to ordering meals from a restaurant, the FA-convenience sample mostly spent less than CAD 25 per person (59%; Supplementary Material Question 14b), and this spending range was more frequent (*p* < 0.05) for this group than for the non-FA sample. The non-FA and FA-general population mostly spent between CAD 25 and 100 per person (51% and 60%, respectively; Supplementary Material Question 14b). In addition, most FA respondents (40% convenience; 63% general) considered that their/their child’s food allergy did not cause them to spend more or less at restaurants than they would do if they did not have a food allergy (Supplementary Material Question 14c).

### 3.6. Selection of FSEs

The criteria applied by the survey participants when deciding which FSE to dine out or order from were explored with a rating question. The same 18 deciding factors were presented to all respondents, whether they were allergic or not. The criteria most frequently ranked at the highest importance level varied per group (Table 5).

As illustrated in Table 5, the non-FA and FA-general population identified “has great-tasting food” as the most important decision factor, and they were also concerned with ingredients’ quality. On the other hand, the FA-convenience sample gave more importance to factors related to their condition. Top choices from both FA respondents also included criteria related to consistency. “Is reasonably priced” was prioritized only by non-FA respondents.

In addition, the rating given by the FA respondents from each group to each of the 18 decision factors (Supplementary Material Question 15) was compared to that of the non-FA respondents. “Ingredients of menu items are readily available”, “willingly accommodates food allergies and dietary observations/restrictions/aversions”, and “allows me to customize my order to meet my needs” were rated higher (*p* < 0.001) on the importance scale by both FA groups compared to the non-FA ones. “Is an establishment I feel safe eating at/ordering food from” and “always gets my order correct” were rated higher (*p* < 0.001) by the FA-convenience sample than by the non-FA ones. “Has generous portion sizes” and “is reasonably priced” were rated higher (*p* < 0.05) by the non-FA respondents than by both FA groups.

As expected, food allergies were more likely (*p* < 0.001) to influence restaurant selection for FA respondents than non-FA ones (“A great deal” and “Some” in Supplementary Material Question 19c; 88% FA-convenience, 74% FA-general, 44% non-allergic). It is noteworthy, however, that for 44% of the non-FA respondents, food allergies had “a great deal” or “some” influence when dining out or ordering food with individuals outside of their household (e.g., friends, extended family, sports team).

### 3.7. Loyalty

**Figure 1:**
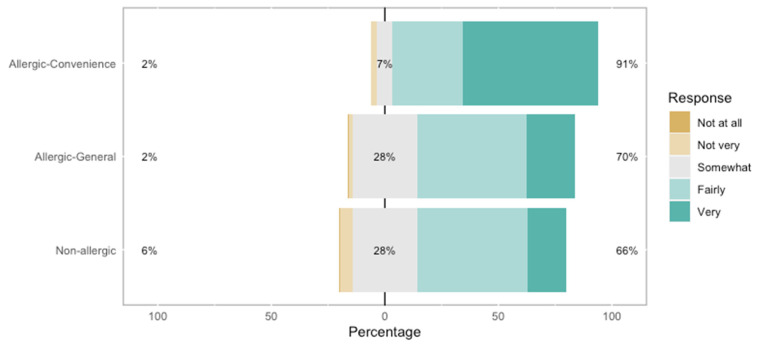
Loyalty to restaurants rated by respondents without food allergies (n = 489), food-allergic-convenience sample (weighted n = 355), and food-allergic-general population (weighted n = 603). Supplementary Material Question 19a.

As shown in Figure 1, FA-convenience sample respondents were more loyal (*p* < 0.001) to restaurants than non-FA ones. The loyalty patterns of non-FA respondents and the FA-general population were not different (*p* > 0.05).

### 3.8. Focus on the Experiences of Allergic Consumers When Dining Out or Ordering Food

FA respondents’ experiences when dining out were further investigated through specific questions. Most FA respondents considered that FSEs do not understand the seriousness of food allergies (82% FA-convenience sample, 71% FA-general population; Supplementary Material Question 17); however, overall, they felt safe when dining out/ordering food (60% convenience, 85% general; Supplementary Material Question 20). Furthermore, most of the FA-general population felt that they had many safe options when dining out/ordering food (“Lots” and “Several” in Supplementary Material Question 20c; 80%), but this was not the case for the FA-convenience sample (“Not very many” and “None” in Supplementary Material Question 20c; 64%).

Among FA respondents who felt safe when dining out/ordering food, reasons related to accommodation and taking precautions (e.g., carrying an epinephrine autoinjector, research, inform server of allergy) were common to both FA groups (Table 6). Among respondents who did not feel safe while dining out/ordering food, lack of accommodation (e.g., careless restaurant workers, inconsistency of establishments) was common to both FA groups (Table 7).

**Figure 2:**
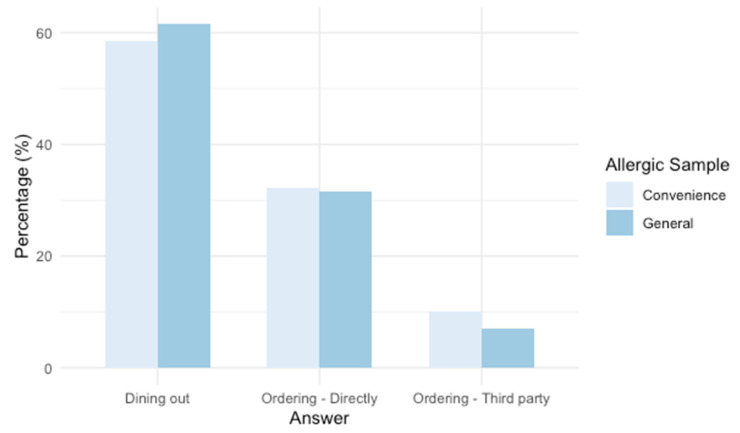
Context of allergic reactions associated with foodservice establishments that occurred within 5 years from the date of the survey, reported by food-allergic-convenience sample respondents (weighted n = 99) and by food-allergic-general population respondents (weighted n = 130). Supplementary Material Question 25a.

**Figure 3:**
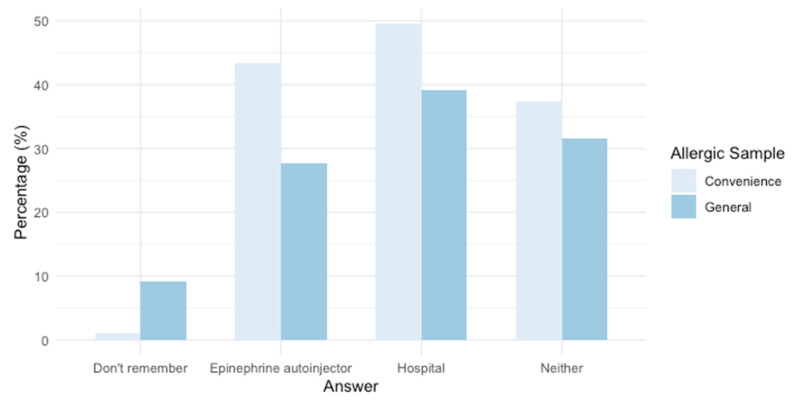
Treatment of allergic reactions associated with FSEs that occurred within 5 years from the date of the survey, reported by food-allergic-convenience sample (weighted n = 99) and food-allergic-general population respondents (weighted n = 130). Supplementary Material Question 25c.

Most of the FA respondents carried an epinephrine autoinjector when dining out (92% FA-convenience sample, 66% FA-general population; Supplementary Material Question 21) and informed the restaurant of their food allergy (90% FA-convenience sample, 57% FA-general population; Supplementary Material Question 22). Nevertheless, some of the FA respondents (43% FA-convenience sample, 27% FA-general population; Supplementary Material Question 23a) had experienced a severe allergic reaction while dining out or as a result of ordering food. Among reactions experienced in the last 5 years in the context of FSEs, most occurred while dining out at a restaurant (58% convenience, 62% general; Figure 2) and the establishment had been informed of the consumer’s allergy (78% convenience, 67% general; Supplementary Material Question 25b). The treatment of these reactions varied; however, most resulted in a trip to the hospital (Figure 3).

Besides allergic reactions, “near misses” (e.g., ordered something that was supposed to be safe but it came with their allergen) were also common among food-allergic respondents (61% FA-convenience sample; 46% FA-general population; Supplementary Material Question 24).

Regarding establishment practices that would be most influential in giving FA participants the assurance needed to dine out/order food comfortably (Supplementary Material Question 30a), “ingredient list is available for all menu items” (44% FA-convenience sample; 33% FA-general population) and “restaurant has a separate and dedicated area to prepare and cook meals for customers with food allergies” (45% FA-convenience sample; 25% FA-general population) were among the top three choices for both groups. Furthermore, with respect to ingredient transparency, FA participants expect restaurants adhering to this practice to provide an ingredient list for all menu items (76% FA-convenience sample; 64% FA-general population; Supplementary Material Question 29) not only for those menu options deemed “allergy suitable”. As expected, the adoption of practices that give FA participants the assurance needed to dine out/order food comfortably (Supplementary Material Question 30a) would result in loyalty to those establishments (94% FA-convenience sample, 83% FA-general population; Supplementary Material Question 30c). A qualitative summary of common behaviors, attitudes, and perceptions among survey respondents discussed so far is presented in Table 8.

Finally, FA participants were questioned about the perceived safety of “vegan” foods (Supplementary Material Question 26). Only a minority of respondents from each group (30% FA-convenience sample; 22% FA-general population) correctly interpreted “vegan” foods as not safe for individuals with milk, egg, shellfish, or fish allergy. Moreover, in both groups, more than 50% interpreted “vegan” items as being safe for individuals with milk, egg, shellfish, or fish allergy, and the majority (67% FA-convenience sample; 55% FA-general population) thought these items were safe for people with a milk allergy.

## 4. Discussion

This study investigated differences in the behaviors, attitudes, and risk perceptions of consumers with (FA-convenience sample, FA-general population) and without food allergies (non-FA) while dining out or ordering meals from FSEs. While sociodemographic differences existed among the survey participants, the responses from FA participants (FA-convenience sample and FA-general population) were weighted with respect to non-FA participants to account for region, gender, age, and household income (Table 1). However, due to inherent differences in their allergy condition, comparisons between the two groups of FA individuals were not attempted. For example, the prevalence of multiple food allergies was higher among the FA-convenience sample than among the FA-general population. This is important in the context of our study as individuals with multiple food allergies adhere to more restricted diets [50]. In the same way, most participants from the FA-convenience sample were diagnosed more than 10 years prior to the survey, and those from the FA-general population were diagnosed within 5 years of the survey’s onset (Table 1). This could imply that the FA-convenience sample is more informed and/or has more experience regarding the management of their condition [51], which could impact their behaviors, attitudes, and risk perceptions towards foods prepared in FSEs. It is therefore important to acknowledge that the inherent differences between the two groups of FA individuals included in this survey may limit the applicability of our findings to the broader allergic population. Thus, to prevent misinterpretation, results grouping both FA populations have been avoided.

Most survey participants from each of the three groups studied dined out or ordered meals from FSEs less than once per week (Table 2). This proportion of avoidance was significantly larger for the FA-convenience sample than for their non-FA counterpart, with the highest avoidance rate noted for ordering through third-party platforms (Table 2). Previous studies have also noted that some FA consumers avoid eating out [13,18,52], suggesting an increased risk perception regarding meals prepared in FSEs. This may be interpreted as a preference among these allergic consumers to have full control over their meal, including its ingredients, preparation, and serving, as a strategy to prevent allergic reactions. Access to this kind of information for meals prepared in FSEs is rare [21] and, if combined with previous negative experiences (e.g., allergic reactions, near misses, perceived lack of accommodation, perceived lack of allergy awareness from FSE staff), could contribute to avoidance.

Regarding the frequency of food consumption prepared in four distinct types of FSEs (Table 3), no significant differences were observed between the non-FA sample and FA-general population. However, respondents from the FA-convenience sample were more likely to avoid each FSE format studied, except full-service chains. As previously reported, consumers with food allergies tend to prefer large chain restaurants due to the availability of online allergen information, consistent menu items, and staff who have undergone more formal training [53]. Moreover, chain restaurant managers tend to apply consistent food safety management practices, which may be more likely to incorporate food allergy training [54,55], ensuring better preparedness among employees to assist customers with food allergies [55]. In addition, the FA-convenience sample’s avoidance of FSEs was more pronounced in restaurants offering a specific type of international cuisine (Table 3). FA consumers’ avoidance of ethnic restaurants is well documented [18,53] and may be explained by the lack of familiarity of FA customers with the diverse ingredients used in these cuisines [30]. Interestingly, the FA-general population reported equivalent or significantly lower avoidance of FSE meals than the non-FA sample (Table 2), and no differences related to type of FSE were noted between these two groups (Table 3).

Most non-FA and FA-general population respondents mentioned cost as a barrier when it comes to dining out or ordering food from an FSE, but not the FA-convenience sample (Table 4 and Table 8). Allergic respondents from both groups also focused on barriers that could compromise their safety (e.g., “Risk of cross-contamination with food allergens”, “Meals are not guaranteed to be safe”) (Table 8). Since survey responses were weighted considering the respondents’ income, statistical correlation with this factor is not possible. However, the fact that the proportion of the FA-convenience sample that saw cost as a barrier was low (≤28%) strongly suggests that they were more affected by safety-related barriers. Also, this group tended to select several barriers, showcasing the challenges they face when considering consuming meals from FSEs. These barriers (Table 4) may also have an impact on this group’s spending range when dining out/ordering food, as they relate not only to restaurant selection but also to the selection of meals within an establishment. Indeed, the FA-convenience sample was less likely to spend more than CAD 100 per person when dining out and more likely to spend less than CAD 25 per person when ordering food than the non-FA sample. This could be attributed to having fewer menu options when managing multiple food allergies (e.g., lack of safe appetizers and/or dessert alternatives). Considering their high income (61% at >CAD 100,000), this finding may also suggest that cost does not play a key role for the FA-convenience sample in the decision to purchase food from an FSE. On the other hand, no differences were noted between the spending ranges of the non-FA sample and FA-general population. Interestingly, most allergic respondents (40% FA-convenience sample; 63% FA-general population) believed that their (or their child’s) food allergy did not result in either more or less spending at restaurants compared to what they would spend if no food allergy was present (Supplementary Material Question 14c).

As expected from the reported barriers, for the FA-convenience sample, factors related to their condition were important when selecting an FSE (i.e., accommodation, feeling of safety, consistency; Table 5). In addition, both FA groups rated ingredient availability, accommodation to allergy and dietary needs, and order customization significantly higher than the non-FA sample. Indeed, a preference by FA consumers for FSEs that can offer these, and other features related to enhanced allergen management, has been reported previously [18,21,33,56]. Although considered a frequent barrier for both the non-FA sample and FA-general population (Table 4), price/cost was among the selection factors with the highest importance rate for the former (Table 5). Other evident non-allergy-related factors were also important for this group (non-FA sample), as expected (i.e., taste, portion size). Nevertheless, food allergies influenced restaurant selection for >40% of non-FA respondents when dining out with individuals outside their immediate household (e.g., friends, extended family, sports teams), highlighting the societal impact of this condition in Canada [57] and the latent market opportunity for FSEs that can meet the expectations of food-allergic customers.

In fact, previous studies report that FA customers return to FSEs that can accommodate them, invest in training, and implement other allergen management strategies [18,33,56], which may translate into loyalty. In this study, increased loyalty to FSEs was reported by the FA-convenience sample when compared to the non-FA sample. No differences were noted between the FA-general population and the non-FA sample. Although loyalty to FSEs may of course be affected by a variety of factors (e.g., service quality, brand image, reputation, customer value, emotional bonding, personal satisfaction) [58,59,60], allergy-related matters are expected to play a role for FA consumers in general [33,53,57,61], as may be deduced from answers to Supplementary Material Questions 13, 15, 20b, 28, 29, and 30a in this study. However, the survey results may exhibit bias towards loyalty as, overall, consumers with food allergies face limited FSE and meal options compared with consumers without food allergies. Given the various factors that can influence consumer loyalty to FSEs, interpretation of our results becomes complex. It can be speculated that, for the food-allergic population, loyalty may be influenced by necessity rather than solely by service quality or other traditional factors that contribute to a positive dining experience. For this group, loyalty to an FSE may therefore be regarded as a strategic choice to mitigate risk. In any case, accommodating consumers with food allergies may indeed offer several advantages for FSEs, including enhanced sales, increased customer appreciation, strengthened customer loyalty [53,61], and brand reputation [29].

Beyond comparison with non-FA participants, this study descriptively explored behaviors, attitudes, and perceptions only relevant to FA individuals. Allergic consumers’ perception that FSEs do not recognize the severity of their condition was widespread (82% FA-convenience sample, 71% FA-general population; Supplementary Material Question 17), aligning with the findings of previous studies [30,53,62]. This may be a result of inadequate practices and/or knowledge of food allergies in Canadian FSEs [31,55,63]. However, most survey participants from both groups of FA individuals felt safe while dining out (60% FA-convenience sample, 85% FA-general population; Supplementary Material Question 20), regardless of the number of options they felt they had when dining out/ordering food. In fact, this may be interpreted in the context of loyalty, where the FA-convenience sample may be recurrently visiting the same few FSEs where they feel safe. For both FA groups, the feeling of safety/not being safe when dining out/ordering food (Table 6, Table 7 and Table 8) seemed to reflect the importance of shared responsibility between foodservice operators, regulators, and FA customers [21,64]. Consumer-related preventive measures, like carrying an epinephrine autoinjector and communicating their food allergies, are crucial to avoiding reactions while dining out, and these were widespread among this study’s participants with food allergies (92% FA-convenience sample, 66% FA-general population; Supplementary Material Question 21). At the same time, FSEs should establish clear communication pathways with consumers and among staff for relaying allergy-related information and strive to accommodate allergy-related requests, as supported by their allergen management practices. Challenges in this area remain, as reported in other jurisdictions [65,66]. Thus, further research is required on food allergen management practices in FSEs and their correlation with staff training [9,17].

Unfortunately, some study participants reported experiencing severe allergic reactions while dining out/ordering food (43% FA-convenience sample, 27% FA-general population; Supplementary Material Question 23a). Reports of allergic reactions associated with FSEs have also been noted in other jurisdictions [9,10,31,67]. The incidence reported in this study underscores the importance of implementing effective allergen management practices in FSEs to protect FA consumers. Furthermore, most reactions reported in this study occurred while dining out in a restaurant (58% FA-convenience sample, 61% FA-general population; Supplementary Material Question 25a; Figure 2), despite informing the establishment of their allergy (78% FA-convenience sample, 67% FA-general population; Supplementary Material Question 25b; Figure 3). Communication of the patron’s allergic condition is crucial and should be further encouraged and facilitated by FSEs [12,13,21,31,62,68]; however, FSEs must complement this strategy with robust allergen management practices in their operations (e.g., training, staff-staff communication, cross-contact prevention, ingredients disclosure) [13,55,62]. Notably, according to allergic respondents, the availability of ingredients for all menu items (44% FA-convenience sample; 33% FA-general population; Supplementary Material Question 30a) and the FSE having a dedicated area to prepare meals for customers with food allergies (45% FA-convenience sample; 25% FA-general population; Supplementary Material Question 30a) would be the most influential practices to provide them with the assurance to dine out/order food comfortably, and foster increased loyalty towards those FSEs (94% FA-convenience sample, 83% FA-general population; Supplementary Material Question 30c). Restaurants that exhibit strong preparedness in addressing food allergy requests can enhance customer experience and promote increased customer loyalty [33]. However, factors such as space limitations, the rapid pace of operations, high employee turnover, frequent changes in recipes, menus [66], and supplied products [29], along with financial considerations [29,65], make the implementation of enhanced allergen management practices challenging for restaurateurs. Furthermore, serving consumers with food allergies presents significant challenges to restaurateurs due to the wide range of potential allergens present in FSEs [69,70,71]. More importantly, a comprehensive regulatory framework for allergen management in foodservice operations is lacking, partly due to the unique challenges posed by these operations compared to the more standardized prepackaged food manufacturing sector [21]. Nevertheless, these legitimate challenges “should not be used as a justification for continued inaction” [34] regarding regulatory requirements associated with allergen management in FSEs and transparency about allergens in the meals prepared in these establishments. The clear expectation for ingredient transparency across all menu items expressed by this study’s participants (76% FA-convenience sample; 64% FA-general population; Supplementary Material Question 29) highlights the impact that regulatory measures on this matter would have for this population.

Apart from allergic reactions, near misses (e.g., ordered something that was supposed to be safe but it came with their child’s/their allergen) were also common (61% FA-convenience sample; 46% FA-general population; Supplementary Material Question 24), suggesting the potential for errors in the context of FSE operations. Multiple steps in the process of preparing and serving a meal in an FSE could result in unintentional allergen presence. A Hazard Analysis Critical Control Points (HACCP)-based approach has been proposed as a risk mitigation strategy [17]; nevertheless, implementation in an FSE context remains challenging. Regardless of the preventive approach adopted, measures targeting training and communication, aiming at accurately communicating risk to allergic consumers and providing sufficient information for them to make an adequate meal selection, are essential [17,21,72].

Unlike prepackaged foods, consumers of non-prepackaged foods often rely on verbal communication to inform their meal choices [13], but regulatory oversight on ingredient declaration in this context is limited [21]. Because of international food labeling standard harmonization for prepackaged foods promoted by the Codex Alimentarius Commission (reference to Codex standard on allergen labeling), most jurisdictions have mandated allergen declaration for prepackaged foods [13,25,27], although the scope and enforcement may vary [21]. In the US, the Food Code provides guidelines for allergen-free meal preparation in FSEs [17,34]. However, only a limited number of FSEs in the US voluntarily disclose allergen information on their menus, and this practice is neither regulated nor standard across all establishments [35]. Canada has not yet adopted specific regulations on allergen declaration for non-prepackaged foods or for allergen management/declaration in FSE settings. Furthermore, allergy communication strategies beyond traditional dine-in settings require attention, especially considering the increase in online food ordering and the use of third-party delivery platforms, where effective communication between the foodservice businesses and their consumers with food allergies is further complicated. Even with the regulation and/or implementation of enhanced allergen management practices, the role played by consumers in the prevention of allergic reactions must not be neglected. For instance, a lack of knowledge in terms of food allergies among FA individuals while dining out or ordering meals from FSEs has been noted [23]. In our study, an important knowledge gap was identified among participants with food allergies, where only a minority in each allergic group (30% FA-convenience sample; 22% FA-general population) correctly identified vegan foods as unsafe for individuals with milk, egg, shellfish, or fish allergies. This result is consistent with previous research [73] reporting that individuals with animal protein allergies often rely on “vegan” labels for safety. Although the term “vegan” generally implies the absence of ingredients of animal origin in the product’s formulation, it is not legally defined as such and it must not be used as an indicator of safety by FA consumers due to the potential presence of cross-contact animal-derived allergens (e.g., milk, eggs, fish, seafood) [73,74,75].

Finally, no significant differences were noted between the responses of the non-FA sample and FA-general population in several questions (Supplementary Material Questions 10; 12; 13; 14a,b; 15; 19a), where differences between non-FA and FA-convenience samples were noted. This study was not designed to allow for statistical comparison between the two groups of FA individuals. Nonetheless, in general terms, it can be said that participants from the FA-convenience sample exhibited greater caution when considering dining out/ordering food, faced challenges when selecting an FSE, and that cost had limited influence on their decision to purchase food from FSEs. This may reflect a potentially higher burden of disease for this subgroup. On the other hand, the behaviors, attitudes, and risk perceptions of the FA-general population when dining out/ordering food more closely resembled those of the non-FA sample, suggesting that FSEs adequately met their allergy needs. The reasons behind this can only be speculated, and may be related, for example, to the participants’ allergen(s), severity of their condition, number of allergies, time since allergy diagnosis, history of allergic reaction in FSEs, and/or access to educational resources, among others. Nevertheless, it is evident that differences within the food-allergic population exist, and generalizations, especially based on studies conducted only with a convenience sample, should be made with caution, as previously noted [41,44,76].

The present study has other limitations apart from those previously mentioned that should be acknowledged. First, the survey was conducted using an online platform, which may have excluded individuals who lacked familiarity with completing internet-based surveys [77]. Also, this study was conducted in Canada, and its results might not be applicable to other jurisdictions. Future research covering other jurisdictions or population segments would allow for the discussion of this study’s findings within a broader scope and are strongly encouraged, both domestically and internationally. Nevertheless, the inclusion of two distinct food-allergic groups (FA-convenience and FA-general population) with allergy diagnosis confirmed by a professional, the comparison of their responses against those from individuals without food allergies, and the high level of representativity obtained represent major strengths of this work.

## 5. Conclusions

Relying on a robust and representative survey, this study described the behaviors, attitudes, and risk perceptions of two distinct food-allergic populations in Canada regarding the consumption of food prepared in FSEs and compared them to those of individuals without food allergies. Our results highlight a potentially higher burden of disease, in terms of psychological and social strain and reduced quality of life, among a subgroup of the food-allergic population (i.e., convenience sample), as reflected in their attitudes and behaviors towards meals prepared in FSEs (e.g., higher avoidance rates, emphasis on safety-related barriers when selecting an FSE or meal). This convenience sample represents members of a food allergy consumer advocacy organization (Food Allergy Canada), who may be more informed and experienced in managing food allergies [51]. Their responses may also reflect characteristics of their food allergy (i.e., number of allergies, years since diagnosis). Nevertheless, both allergic groups (those affiliated and not affiliated with Food Allergy Canada) expressed shared concerns and needs regarding their safety when consuming food prepared in FSEs (Table 8). These concerns should be considered by FSEs and regulators when designing risk management strategies. Notably, the disclosure of ingredients for all menu items would significantly contribute to the effective protection of these groups and enhance their overall perception of safety when dining out or ordering food. In addition, implementing measures to prevent allergen cross-contact in FSEs and establishing clear communication processes for allergy-related food orders would provide a solid foundation for improving food allergy safety in FSE settings.

## Figures and Tables

**Table 1:** Summary of sociodemographic and food allergy characteristics of survey respondents.

Variable	No Food Allergies	Food-Allergic	
		Convenience	General
Sample size	500	500	500
Weighted sample size	N/A	390	610
Age (most common)	35–54 years (41%)	35–54 years (47%)	35–54 years (37%)
Income (most common)	<CAD 100,000 (52%)	>CAD 100,000 (61%)	<CAD 100,000 (62%)
	F (50%); M (49.9%); Other/no answer (0.1%)	F (72%); M (26%); Other/no answer (2%)	F (35.8%); M (63.9%); Other/no answer (0.3%)
Respondent status	N/A	Adult with food allergy (57%); Parent of child with food allergy (43%)	Adult with food allergy (68%); Parent of child with food allergy (32%)
Date of food allergy diagnosis (most common) ^1^		>10 years from survey (59%)	Within 5 years from survey (42%)
Diagnosed by ^1^		Allergist/immunologist (86%); Family physician (25%)	Allergist immunologist (51%); Family physician (53%)
Number of allergies (most common) ^1^		Multiple (75%)	Single (56%)
Allergen (most common) ^1^		Peanuts (61%); Tree nuts (61%)	Peanuts (35%); Tree nuts (26%)
Allergen (least common)		Mustard (4%)	Mustard (3%)
Prescribed epinephrine autoinjector (most common) ^1^		Yes (93%)	Yes (54%)

^1^ Differences between the 2 groups of FA individuals: FA-convenience sample (i.e., members of Food Allergy Canada’s network) and FA-general population (i.e., recruited through an external panel).

**Table 2:** Frequency of consumption of food prepared in FSEs. For each service format, frequencies from food-allergic respondents that were significantly different (*p* < 0.05) from frequencies from respondents without food allergies are marked with a (*). Supplementary Material Question 10.

Service Format	Frequency	No Food Allergies ^1^	Food-Allergic	
			Convenience ^2^	General ^3^
Dining in a restaurant	≥1× week	27%	14% (*)	26%
	<1× week ^4^	68%	73%	72%
	Never	5%	13% (*)	2% (*)
Ordering take-out or delivery directly from a restaurant	≥1× week	34%	19% (*)	35%
	<1× week ^4^	59%	61%	57%
	Never	7%	20% (*)	8%
Ordering delivery through a 3rd-party service	≥1× week	20%	6% (*)	29% (*)
	<1× week ^4^	45%	28% (*)	46%
	Never	35%	66% (*)	25% (*)

^1^ Sample size = 500; ^2^ weighted sample size = 390; ^3^ weighted sample size = 610; ^4^ excludes the answer choice “Never”.

**Table 3:** Respondents’ frequency of consumption of food prepared in 4 distinct types of FSEs. For each establishment type, frequencies from food-allergic respondents that were significantly different (*p* < 0.05) from frequencies from respondents without food allergies are marked with a (*). Supplementary Material Question 12.

Establishment Type	Frequency	No Food Allergies ^1^	Food-Allergic	
			Convenience ^2^	General ^3^
Fast food/quick service	≥1× week	41%	21% (*)	41%
	<1× week	57%	63%	56%
	Never	2%	16% (*)	3%
Full-service chains	≥1× week	10%	6% (*)	11%
	<1× week	78%	80%	79%
	Never	12%	14%	10%
Independent (e.g., smaller, privately owned restaurants)	≥1× week	17%	7% (*)	18%
	<1× week ^4^	77%	74%	77%
	Never	6%	19% (*)	5%
Specific type of international cuisine	≥1× week	14%	4% (*)	15%
	<1× week ^4^	78%	52% (*)	75%
	Never	8%	44% (*)	10%

^1^ Sample size = 489; ^2^ weighted sample size = 355; ^3^ weighted sample size = 603; ^4^ excludes the answer choice “Never”.

**Table 4:** Most common barriers when dining out/ordering food from a foodservice establishment. Supplementary Material Question 13.

	No Food Allergies ^1^	Food-Allergic	
		Convenience ^2^	General ^3^
**When dining out**			
Barrier #1	Cost/too expensive (65%)	Risk of cross-contamination with food allergens (79%)	Cost/too expensive (45%)
Barrier #2	Prefer home-cooked meals (30%)	Lack of access to ingredient information for menu items (73%)	Risk of cross-contamination with food allergens (37%)
Barrier #3	Not convenient (24%)	Meals are not guaranteed to be safe (69%)	My child has had/I have had an allergic reaction in the past (35%)
**When ordering food**			
Barrier #1	Cost/too expensive (60%)	Risk of cross-contamination with food allergens (73%)	Cost/too expensive (43%)
Barrier #2	Prefer home-cooked meals (25%)	Lack of access to ingredient information for menu items (66%) Not confident the restaurant will understand my child’s/my specific food allergy (66%)	Risk of cross-contamination with food allergens (38%)
Barrier #3	Dining out/ordering food from a restaurant is unhealthy (23%)	Meals are not guaranteed to be safe (65%)	Meals are not guaranteed to be safe (36%)

^1^ Sample size = 500; ^2^ weighted sample size = 390; ^3^ weighted sample size = 610.

**Table 5:** Factors are most frequently ranked at the highest levels of importance when deciding which foodservice establishment to dine out or order from. Supplementary Material Question 15.

	No Food Allergies ^1^	Food-Allergic	
		Convenience ^2^	General ^3^
Factor #1	Has great-tasting food (82%)	Willingly accommodates food allergies and dietary observations/restrictions/aversions (98%)	Has great-tasting food (79%)
Factor #2	Is reasonably priced (77%)	Is an establishment I feel safe eating at/ordering food from (97%)	Always gets my order correct (77%)
Factor #3	Uses fresh, good-quality ingredients (73%)	Is always consistent (89%)	Uses fresh, good-quality ingredients (75%)

^1^ Sample size = 489; ^2^ weighted sample size = 355; ^3^ weighted sample size = 603.

**Table 6:** Food-allergic respondents’ top reasons for feeling safe while dining out/ordering food. Supplementary Material Question 20b.

	Food-Allergic	
	Convenience ^1^	General ^2^
Reason #1	Communication/staff’s ability to communicate (24%)	Accommodation/willingness to accommodate (20%)
Reason #2	Accommodation/willingness to accommodate (23%) Prefer a familiar/trusted restaurant (23%)	Taking precautions (19%)
Reason #3	Taking precautions (21%)	Type of allergy (12%)

^1^ Weighted sample size = 233; ^2^ weighted sample size = 521.

**Table 7:** Food-allergic respondents’ top reasons for not feeling safe while dining out/ordering food. Supplementary Material Question 20b.

	Food-Allergic	
	Convenience ^1^	General ^2^
Reason #1	Lack of accommodation (40%)	Lack of accommodation (21%)
Reason #2	Negative previous experiences (21%)	Lack of trust/fear (20%)
Reason #3	Communication/staff’s ability to communicate (19%)	Ability to guarantee safety (19%)

^1^ Weighted sample size = 157; ^2^ weighted sample size = 89.

**Table 8:** Qualitative summary of common behaviors, attitudes, and perceptions among survey respondents.

	No Food Allergies	Food-Allergic	
		General	Convenience
Barriers to dining out or ordering food			
Cost	✓	✓	
Risk of allergen cross-contamination	N/A	✓	✓
Meals are not guaranteed to be safe		✓	✓
FSE selection factors			
Taste	✓	✓	
Ingredient quality	✓	✓	
Consistency, serving correct order		✓	✓
Availability of ingredient information		✓	✓
Willingness to accommodate		✓	✓
Perceptions when dining out or ordering food			
FSEs do not understand seriousness of food allergy	N/A	✓	✓
Feel safe when dining out		✓	✓
There are many safe options		✓	
Feeling of safety when dining out or ordering food			
Willingness to accommodate (FSE)	N/A	✓	✓
Taking precautions (consumer with food allergy)		✓	✓
Availability of ingredient information (FSE)		✓	✓
Dedicated area to prepare meals for consumers with food allergy (FSE)		✓	✓

## Data Availability

The aggregated, anonymous data that support the findings presented in this study are available upon request due to ethical reasons.

## Supplementary Materials

The following supporting information can be downloaded at https://www.mdpi.com/article/10.3390/nu17182916/s1, Supplementary Material: Questionnaire.

## Abbreviations

The following abbreviations are used in this manuscript:

FSEs	Foodservice establishments
FA	Food-allergic
