# Supplementary material for: Food Allergy and Foodservice: A Comparative Study of Allergic and Non-Allergic Consumers’ Behaviors, Attitudes, and Risk Perceptions
Source: Nutrients. 2025 Sep 9;17(18):2916. doi: 10.3390/nu17182916 (PMC12477836; doi:10.3390/nu17182916)
Supplement: Supplementary file 1 [file nutrients-17-02916-s001.zip › nutrients-3843855-supplementary.pdf]

STRATEGIC Navigator

FAC Informed Dining Strategy

Questionnaire (FAC-1626)

DRAFT: October 4, 2023

Project Specs

Methodology: Online survey using FAC's database & a third-party panel.

Geographic scope: National, including Quebec.

Sample size: 1500 completes, broken out as follows:

- 500 completes sourced from FAC's database
- 500 completes with **food-allergic consumers** sourced through a third-party panel, broken out as follows:
    - 60 completes in BC.
    - 80 completes in Prairies
    - 190 completes in Ontario
    - 120 completes in Quebec
    - 50 completes in Atlantic Canada

  [IN ANALYSIS PHASE WEIGHT REGIONS AS FOLLOWS: 13% BC, 17% Prairies, 39% Ontario, 24% Quebec, 7% Atlantic]

- 500 completes with **<u>NON</u>-food-allergic consumers** sourced through a third-party panel, broken out as follows:
    - 60 completes in BC.
    - 80 completes in Prairies
    - 190 completes in Ontario
    - 120 completes in Quebec
    - 50 completes in Atlantic Canada

  [IN ANALYSIS PHASE WEIGHT REGIONS AS FOLLOWS: 13% BC, 17% Prairies, 39% Ontario, 24% Quebec, 7% Atlantic]

Qualifiers:

- Q1 (panel respondents) – Must reside in BC, Prairies, Ontario, Quebec, or Atlantic Canada – Watch quotas.
- Q2 — Must be 18+
- Q3 – Must have a food allergy or have a child with a food allergy
- Q5– Food allergy was diagnosed / confirmed by a medical professional (pediatrician, family physician (GP), allergist, emergency room doctor, etc.)

Questionnaire

SECTION 1: Screener & Profiling

1. In which province or territory do you currently reside?

British Columbia	❒
Alberta	❒
Manitoba	❒
Saskatchewan	❒
Ontario	❒
Quebec	❒
Newfoundland & Labrador	❒
Nova Scotia	❒
New Brunswick	❒
Prince Edward Island	❒
Nunavut	❒
Yukon	❒
Northwest Territories	❒
None of the above	❒

[PANEL RESPONDENTS MUST RESIDE IN BC, PRAIRIES, ONTARIO, QUEBEC OF ATLANTIC CANADA; OTHERWISE THANK & TERMINATE]
[WATCH QUOTAS FOR PANEL RESPONDENTS]

[TERMINATION MESSAGE FOR PANEL RESPONDENTS TO READ: Thank you for your interest. Unfortunately, this survey is only applicable to those living in BC, the Prairies, Ontario, Quebec of Atlantic Canada]

2. To ensure we reach a wide cross-section of people, into which of the following age ranges do you fall?

Under 18	❒	**[THANK & TERMINATE]**
18 to 24	❒	
25 to 34	❒	
35 to 44	❒	
45 to 54	❒	
55 to 64	❒	
65 or older	❒	
**[SHOW TO FAC DATABASE ONLY]** Prefer not to answer	❒	

[TERMINATION MESSAGE TO READ: Thank you for your interest. Unfortunately, this survey is only open to adults 18 years of age or older.]

3. Which of the following best describes you?

[ALLOW RESPONDENTS TO CHECK ONE OR BOTH OF THE FIRST 2 OPTIONS OR THE THIRD OPTION]

I have a food allergy	❑	
I am a parent of a child with a food allergy	❑	
I do not have any food allergies nor do any of my children	❑	**[THANK & TERMINATE IF FROM <u>FAC DATABASE</u>]**

[IF FROM FAC DATABASE AND CHECK 'I do not have any food allergies no do any of my children' THANK & TERMINATE. TERMINATION MESSAGE SHOULD READ: Thank you for you interest in this survey. Unfortunately the questions are only relevant to those with a food allergy or parents of a child with a food allergy.]

4. RESPONDENT CLASSIFICATION – DO NOT ASK

IF 'I have a food allergy' AT Q3 CLASSIFY AS AN <u>ADULT (with a food allergy)</u>

IF 'I am a parent of a child with a food allergy' AT Q3 CLASSIFY AS A <u>PARENT (of a child with a food allergy)</u>

IF RESPONDENT CHECKS BOTH 'I have a food allergy' AND 'I am a parent of a child with a food allergy' CLASSIFY <u>AS AN ADULT (with a food allergy).</u>

IF RESPONDENT CHECKS 'I do not have any food allergies nor do any of my children'

CLASSIFY AS A <u>NON-FOOD ALLERGIC CONSUMER.</u>

[IF CLASSIFIED AS AN 'Adult' SHOW] We would like you to answer the remainder of this survey based on your experiences and needs as an **adult** with a food allergy.

[IF CLASSIFIED AS A 'PARENT' SHOW] We would like you to answer the remainder of this survey based on your experiences and needs as a **<u>parent of a child</u>** with a food allergy. If you have **more than one child with a food allergy**, please complete the survey **based on the child whose birthday is coming up next**. In the case of twins, answer for the older twin.

5. **[IF 'Parent' AT Q4]** Who diagnosed your child's <u>food</u> allergy? If you have more than one child with a food allergy, please answer based on the child whose birthday is coming up next. In the case of twins, answer for the older twin.

[IF 'Adult' AT Q4] Who diagnosed your <u>food</u> allergy?

(Select ALL that apply)

I made the diagnosis myself	❑	
Allergist / Immunologist	❑	**[MUST SELECT AT LEAST ONE OF THESE 4 OPTIONS; OTHERWISE THANK & TERMINATE]**
Family Physician	❑	
Emergency Department Physician	❑	
Pediatrician		
Gastroenterologist	❑	
Other (please specify)________________	❑	

[TERMINATION MESSAGE TO READ: Thank you for your interest. Unfortunately, this survey is only applicable to those who have had a formal food allergy diagnosis.

6. **[IF 'Parent' AT Q4 ASK]** What is the age of your child with a food allergy? Again, if you have more than one child with a food allergy, please answer based on the child whose birthday comes next.

5 years of age or younger	❑
6 to 9	❑
10 to 12	❑
13 to 17	❑
18 years of age or older	❑

7. **[IF 'Parent' AT Q4 ASK]** Which **<u>food(s)</u>** is your child allergic to?

[IF 'Adult' AT Q4 ASK] Which **<u>food(s)</u>** are you allergic to?

(Select ALL that apply)

[SHOW IN ALPHABETICAL ORDER]

Egg	❑
Fish (e.g., trout, salmon)	❑
Milk	❑
Mustard	❑
Peanut	❑
Sesame	❑
Shellfish - Molluscs (such as oysters, mussels, squid)	❑
Shellfish - Crustaceans (such as lobster, shrimp, crab)	❑
Soy	❑
Sulphites (an additive, not a food)	❑
Tree nuts (e.g., almonds, Brazil nuts, cashews, hazelnuts, macadamia nuts, pecans, pine nuts, pistachios, walnuts)	❑
Wheat & triticale	❑
**[ALWAYS SHOW LAST]** Other foods (please specify)___________________	❑

8. **[IF 'Parent' AT Q4 ASK]** When was your child first diagnosed with their food allergy? If they have more than one food allergy, please answer based on the allergy that was first diagnosed.

Less than 6 months ago	❑
6 to 11 months ago	❑
1 to 2 years ago	❑
3 to 5 years ago	❑
6 to 10 years ago	❑
More than 10 years ago	❑

[IF 'Adult' AT Q4 ASK] When were you first diagnosed with your food allergy? If you have more than one food allergy, please answer based on the allergy that was first diagnosed.

Less one year ago	❑
1 to 5 years ago	❑
6 to 10 years ago	❑
More than 10 years ago	❑

9. **[IF 'PARENT' AT Q4 ASK]** Has your child been prescribed an epinephrine auto-injector (e.g., EpiPen®, ALLERJECT®, Emerade®)?

[IF 'Adult' AT Q4 ASK] Have you been prescribed an epinephrine auto-injector (e.g., EpiPen®, ALLERJECT®, Emerade®)?

Yes	❑
No	❑

SECTION 2: Food Service Behaviour & Attitudes

10. How often, if ever, do you do the following?

**[RANDOMIZE]**	Multiple times per week	Once a week	Every 2 to 3 weeks	Once a month	Every 2 to 3 months	Less often than every 3 months	Never
**[FOR PARENTS SHOW]** Dine in a restaurant with your child **[FOR ADULTS OR NON-FOOD ALLERGIC CONSUMERS SHOW]** Dine in a restaurant	❑	❑	❑	❑	❑	❑	❑
**[FOR PARENTS SHOW]** Order food through take-out/delivery directly from a restaurant where at least part of the order will be consumed by your child. **[FOR ADULTS OR NON-FOOD ALLERGIC CONSUMERS SHOW]** Order food through take-out/delivery directly from a restaurant.	❑	❑	❑	❑	❑	❑	❑
**[FOR PARENTS SHOW]** Order food through a third-party delivery service / app (e.g., Skip, Uber Eats) where at least part of the order will be consumed by your child **[FOR ADULTS OR NON-FOOD ALLERGIC CONSUMERS SHOW]** Order food through a third-party delivery service / app (e.g., Skip, Uber Eats)	❑	❑	❑	❑	❑	❑	❑

[FOR PARENTES & ADULTS SHOW] This survey is going to focus on food allergies as they relate to dining out and / or ordering food from a restaurant or food service establishment.

[IF PARENT AT Q4 ADD] Please answer the questions based on your experiences and needs as a **parent of a child** with a food allergy.

[FOR NON-FOOD ALLERGIC CONSUMERS SHOW] This survey is going to focus on dining out and / or ordering food from a restaurant or food service establishment.

**[SHOW TO ALL]**<br><br>**IMPORTANT – PLEASE READ:**<br><br>Throughout this survey, when we say **dine out**, we mean **any type** of restaurant or foodservice establishment (e.g., fast food / quick service restaurants, full-service restaurants, restaurant chains, independent / privately owned restaurants, etc.)<br><br>When we say **order food from**, this would include both **take-out** or **delivery** orders, including those placed directly through the restaurant and those placed through a third-party delivery service or app.

11. **[IF 'Never' TO ALL AT Q10 ASK]** In the previous question you indicated that you do not dine out or order food from restaurants or foodservice establishments. Why is that? Please be as specific and detailed as possible.

12. **[DO NOT ASK IF "Never" FOR ALL AT Q10]** How often do you dine out at or order food from the following types of restaurants / foodservice establishments?

[IF PARENT ADD] When answering, please **only include occasions where your child with the food allergy is with you or will be consuming the food order.**

**[RANDOMIZE]**	**Once a week or more**	**Every 2 to 3 weeks**	**Once a month**	**Every 2 to 3 months**	**Less often than every 3 months**	**Never**
Fast food / quick service restaurants (e.g., McDonald's, Tim Horton's, Subway, Pizza Pizza, Starbucks)	❑	❑	❑	❑	❑	❑
Full-service chains (e.g., Kelsey's Earls, Moxies, The Keg, Milestones, Swiss Chalet)	❑	❑	❑	❑	❑	❑
Independent restaurants (e.g., smaller, privately owned restaurants)	❑	❑	❑	❑	❑	❑
Restaurants specializing in a specific type of international cuisine (e.g., Thai, Indian, Sushi, Chinese)	❑	❑	❑	❑	❑	❑

13. Which of the following, if any, represent barriers for you when it comes to dining out / ordering food from a restaurant or foodservice establishment or doing so more often?

(For each column, select ALL that apply)

**[RANDOMIZE]**	**Barriers When <u>Dining Out</u>**	**Barriers When <u>Ordering</u> Food** (Directly From Restaurant or Through Delivery Service / App)
Cost / too expensive	❑	❑
Not convenient	❑	❑
Prefer home cooked meals	❑	❑
Dining out / ordering food from a restaurant is unhealthy		
Members of my household can never agree on a restaurant		
I am a picky eater / I have picky eaters	❑	❑
Lack of access to ingredient information for menu items	❑	❑
**[FOR PARENTS SHOW] Don't** like asking for special accommodations because of my child's food allergy **[FOR ADULTS SHOW]** Don't like asking for special accommodation because of my food allergy **[FOR NON-FOOD ALLERGIC SHOW]** Don't like to ask for special accommodation because of my dietary needs / observances	❑	❑
**[FOR PARENTS SHOW]** Don't like to be made to feel different or bothersome because of my child's food allergy **[FOR ADULTS SHOW]** Don't like to be made to feel different or bothersome because of my food allergy **[FOR NON-FOOD ALLERGIC SHOW]** Don't like to be made to feel different of bothersome because of my dietary needs / observances	❑	❑
**[DO <u>NOT</u> SHOW TO NON-FOOD ALLERGIC CONSUMERS]** Risk of cross-contamination with food allergen(s)	❑	❑
**[DO <u>NOT</u> SHOW TO NON-FOOD ALLERGIC CONSUMERS]** Stress related to conveying food allergy to restaurant staff	❑	❑
**[DO <u>NOT</u> SHOW TO NON-FOOD ALLERGIC CONSUMERS]**	❑	❑
**[DO <u>NOT</u> SHOW TO NON-FOOD ALLERGIC CONSUMERS]** Not worth the risk of an allergic reaction	❑	❑
**[DO <u>NOT</u> SHOW TO NON-FOOD ALLERGIC CONSUMERS]** Lack of consistent practices / policies between foodservice establishments in terms of how they manage food allergies	❑	❑

**[DO <u>NOT</u> SHOW TO NON-FOOD ALLERGIC CONSUMERS]** Most restaurants do not take food allergies seriously enough / do not understand the seriousness of food allergies	❑	❑
**[DO <u>NOT</u> SHOW TO NON-FOOD ALLERGIC CONSUMERS]** It is too difficult to find safe food options	❑	❑
**[DO NOT SHOW TO NON-FOOD ALLERGIC CONSUMERS]**<br>**FOR PARENTS SHOW:** Not confident the restaurant will understand my child's specific food allergies and communicate it properly to those preparing the food<br>FOR ADULTS SHOW: Not confident the restaurant will understand my specific food allergies and communicate it properly to those preparing the food	❑	❑
**[DO <u>NOT</u> SHOW TO NON-FOOD ALLERGIC CONSUMERS]**<br>Meals are not guaranteed to be safe	❑	❑
**[DO <u>NOT</u> SHOW TO NON-FOOD ALLERGIC CONSUMERS]**<br>**[FOR PARENTS SHOW]** My child has had an allergic reaction in the past<br>**[FOR ADULTS SHOW]** I have had an allergic reaction in the past	❑	❑
**[DO <u>NOT</u> SHOW TO NON-FOOD ALLERGIC CONSUMERS]**<br>**[FOR PARENTS SHOW]** Too many "near misses" (e.g., ordered something that was supposed to be safe and it came with my child's allergen)<br>**[FOR ADULTS SHOW]** Too many "near misses" (e.g., ordered something that was supposed to be safe and it came with my allergen)	❑	❑
**[ALWAYS SHOW 2nd LAST]** Other (please specify)____________	❑	❑
**[ALWAYS SHOW LAST]** None of the above	❑	❑

[SHOW Q14a & Q14b ON SAME SCREEN AS APPROPRIATE]

14a. **[DO NOT ASK IF "Never" TO DINE OUT Q10]** Approximately how much do you typically spend per person when dining out at a restaurant or foodservice establishment?

[FOR PARENT ADD] Please consider occasions where your child would be present or consuming the order.

Less than $25	❑
$25 to $50	❑
$51 to $100	❑
$101 to $150	❑
$151 to $200	❑
More than $200	❑

14b. **[DO NOT ASK IF "Never" FOR BOTH ORDER FOOD DIRECTLY THROUGH RESTAURANT AND ORDER FOOD THROUGH THIRD-PARTY APP Q10]** Approximately how much do you typically spend per person when ordering food from a restaurant or foodservice establishment? This would include take-out or delivery orders and those ordered directly from the restaurant or those ordered through a third-party food service delivery service / app.

[FOR PARENT ADD] Please consider occasions where your child would be present or consuming the order.

Less than $25	❑
$25 to $50	❑
$51 to $100	❑
$101 to $150	❑
$151 to $200	❑
More than $200	❑

14c. **[IF PARENT & ANSWERED Q14a OR Q14b ASK]** Does your child's food allergy cause you to spend more, the same amount or less at restaurants than you would if they did not have a food allergy?

[IF ADULT & ANSWERED Q14a OR Q14b ASK] Does your food allergy cause you to spend more, the same amount or less at restaurants than you would if you did not have a food allergy?

More	❑
Same amount	❑
Less	❑
Don't know	❑

15. **[DO <u>NOT</u> ASK IF "Never" FOR ALL AT Q10]** How important are the following factors when deciding which restaurant / foodservice establishment to dine out at or order food from?
[FOR PARENTS ADD] Please base your responses on foodservice occasions your child is a part of.

**[RANDOMIZE]**	**1 – Not at all important**	**2**	**3**	**4**	**5**	**6**	**7**	**8**	**9**	**10 – Very important**
Is conveniently located	❑	❑	❑	❑	❑	❑	❑	❑	❑	❑
Has great tasting food	❑	❑	❑	❑	❑	❑	❑	❑	❑	❑
Uses fresh, good quality ingredients										
Has good customer service	❑	❑	❑	❑	❑	❑	❑	❑	❑	❑
Offers something for everyone	❑	❑	❑	❑	❑	❑	❑	❑	❑	❑
Is always consistent	❑	❑	❑	❑	❑	❑	❑	❑	❑	❑
Is reasonably priced	❑	❑	❑	❑	❑	❑	❑	❑	❑	❑
Willingly accommodates food allergies, dietary observations / restrictions / aversions	❑	❑	❑	❑	❑	❑	❑	❑	❑	❑
Gets good reviews / is recommended by others	❑	❑	❑	❑	❑	❑	❑	❑	❑	❑
Is a restaurant I've never tried before	❑	❑	❑	❑	❑	❑	❑	❑	❑	❑
Has an appealing vibe / ambiance	❑	❑	❑	❑	❑	❑	❑	❑	❑	❑
Staff are friendly and welcoming	❑	❑	❑	❑	❑	❑	❑	❑	❑	❑
Is an establishment I feel safe eating at / ordering food from	❑	❑	❑	❑	❑	❑	❑	❑	❑	❑
Always gets my order correct	❑	❑	❑	❑	❑	❑	❑	❑	❑	❑
Food is prepared in a timely manner	❑	❑	❑	❑	❑	❑	❑	❑	❑	❑
Ingredients of menu items are readily available	❑	❑	❑	❑	❑	❑	❑	❑	❑	❑
Has generous portion sizes	❑	❑	❑	❑	❑	❑	❑	❑	❑	❑
Allows me to customize my order to meet my needs	❑	❑	❑	❑	❑	❑	❑	❑	❑	❑

16. **[ASK TO PARENTS & ADULTS ONLY]** If a restaurant claims to be "allergy-friendly", what does that mean to you? What would your expectation be?

17. **[ASK TO PARENTS & ADULTS ONLY]** To what extent do you agree or disagree with the following statement?

**Most restaurants and foodservice establishments do not understand the seriousness of food allergy.**

Strongly agree ❑

Somewhat agree ❑

Somewhat disagree ❑

Strongly disagree ❑

18. **[ASK TO PARENTS & ADULTS ONLY; DO NOT ASK IF "Never" FOR ALL AT Q10]** How often, if ever, do you choose specific restaurants because of their allergen policy or availability of ingredient information for menu items?

Always ❑

Often ❑

Occasionally ❑

Rarely ❑

Never ❑

19a. **[IF 'PARENT' AT Q4 ASK & NOT "Never" FOR ALL AT Q10]** When it comes to dining out with or ordering food from a restaurant for your child, how loyal would you say you are? Do you typically visit or order from the same place(s) or are you always trying new places?

[IF 'ADULT' OR 'NON-FOOD ALLERGIC CONSUMER' AT Q4 ASK & NOT "Never" FOR ALL AT Q10] When it comes to dining out or ordering food from a restaurant, how loyal would you say you are? Do you typically visit or order from the same place(s) or are you always trying new places?

Very loyal ❑
Fairly loyal ❑
Somewhat loyal ❑
Not very loyal ❑
Not at all loyal ❑

19b. **[IF 'PARENT' AT Q4 ASK & NOT "Never" FOR ALL AT Q10]** How much influence does your child's food allergy have on your level of restaurant loyalty?

[IF 'ADULT' AT Q4 ASK & NOT "Never" FOR ALL AT Q10] How much influence does your food allergy have on your level of restaurant loyalty?

- A great deal of influence ❑
- Some influence ❑
- Not very much influence ❑
- No influence ❑

19c. **[IF 'PARENT' AT Q4 ASK & NOT "Never" ALL AT Q10]** When dining out or ordering food with those outside your immediate household (e.g., friends, extended family, sports teams), how much influence, if any, does your child's food allergy have on the restaurant selection?

[IF 'ADULT' AT Q4 ASK & NOT "Never" FOR ALL AT Q10] When dining out or ordering food with those outside your immediate household (e.g., friends, extended family, sports teams), how much influence, if any, does your food allergy have on the restaurant selection?

[IF 'NON-FOOD ALLERGIC CONSUMER' & NOT "Never" FOR ALL AT Q10] When dining out or ordering food with those outside your immediate household (e.g., friends, extended family, sports teams), how much influence, if any, do food allergies have on the restaurant selection?

- A great deal of influence ❑
- Some influence ❑
- Not very much influence ❑
- No influence ❑

[SHOW Q20a & Q20b ON SAME SCREEN]

20a. **[IF 'PARENT' AT Q4 ASK]** Overall, how safe do you feel dining out / ordering food from a restaurant or foodservice establishment given your child's food allergy?

[IF 'Adult' AT Q4 ASK] Overall, how safe do you feel dining out / ordering food from a restaurant or foodservice establishment given your food allergy?

- Very safe ❑
- Somewhat safe ❑
- Not very safe ❑
- Not at all safe ❑

20b. Please explain your response above in as much detail as possible, including what contributes to feeling safe or unsafe.

20c. [IF **'PARENT' AT Q4 ASK]** When it comes to your child's food allergy and dining out / ordering food, do you feel you have…

[IF 'ADULT' AT Q4 ASK] When it comes to your food allergy and dining out / ordering food, do you feel you have…

- Lots of safe options ❑
- Several safe options ❑
- Not very many safe options ❑
- No safe options ❑

21. **[IF A PARENT AT Q4, 'Yes' AT Q9 AND <u>NOT</u> NEVER FOR DINING OUT AT Q10]** How often, if ever, does your child have their epinephrine auto-injector with them when dining out? This would include instances where you may carry their auto-injector for them.

[IF ADULT AT Q4, 'Yes' AT Q9 AND <u>NOT</u> NEVER FOR DINING OUT AT Q10] How often, if ever, do you have your epinephrine auto-injector with you when dining out?

- Always ❑
- Most of the time ❑
- Occasionally ❑
- Sometimes ❑
- Never ❑

22a. **[IF A PARENT AT Q4, 'Yes' AT Q9 AND <u>NOT</u> 'Never' FOR ALL AT Q10 ASK]** When dining out or ordering food from a restaurant or foodservice establishment, how often, if ever, do you inform the restaurant of your child's food allergy?

[IF ADULT AT Q4, 'Yes' AT Q9 AND <u>NOT</u> 'Never' FOR ALL AT Q10 ASK] When dining out or ordering food from a restaurant or foodservice establishment, how often, if ever, do you inform the restaurant of your food allergy?

Always	❑
Most of the time	❑
Occasionally	❑
Sometimes	❑
Never	❑
Do not dine out	❑

22b. **[IF PARENT & 'Most of the time', 'Occasionally', 'Sometimes' or 'Never' AT Q22a ASK]** When dining out or ordering food why don't you always inform the restaurant of your child's food allergy? What contributes to not disclosing the allergy?

[IF ADULT & 'Most of the time', 'Occasionally', 'Sometimes' or 'Never' AT Q22a ASK] When dining out or ordering food why don't you always inform the restaurant of your food allergy? What contributes to not disclosing the allergy?

23a. **[IF A PARENT AT Q4 AND <u>NOT</u> 'Never' FOR ALL AT Q10 ASK ASK]** Has your child <u>ever</u> experienced a severe allergic reaction while dining out or as a result of ordering food from a restaurant or foodservice establishment?

[IF ADULT AT Q4 AND <u>NOT</u> 'Never' FOR ALL AT Q10 ASK ASK] Have you <u>ever</u> experienced a severe allergic reaction while dining out or as a result of ordering food from a restaurant or foodservice establishment?

Yes	❑
No	❑

23b. **[IF PARENT AND 'Yes' AT Q23 ASK]** How recently has your child experienced a severe allergic reaction while dining out or as a result of ordering food from a restaurant or foodservice establishment?

[IF ADULT "Yes' AT Q23 ASK] How recently have you experienced a severe allergic reaction while dining out or as a result of ordering food from a restaurant or foodservice establishment?

Within the past 6 months	❑
Within the past year	❑
Within the past 2 years	❑
Within the past 3 to 5 years	❑
Last allergic reaction was more than 5 years ago	❑

24. **[IF A PARENT AT Q4 AND NOT 'Never' FOR ALL AT Q10 ASK ASK]** Has your child ever experienced a "near miss" (e.g., ordered something that was supposed to be safe and it came with your child's allergen) while dining out or as a result of ordering food from a restaurant or foodservice establishment?

[IF ADULT AT Q4 AND NOT 'Never' FOR ALL AT Q10 ASK ASK] Have you ever experienced a "near miss" (e.g., ordered something that was supposed to be safe and it came with your allergen) while dining out or as a result of ordering food from a restaurant or foodservice establishment?

Yes	❑
No	❑

[SHOW Q25a, Q25b & Q25c ON SAME SCREEN – ONLY ASK TO PARENTS & ADULTS WHO CHECK FIRST 4 OPTIONS AT Q23b]

[IF PARENT & WITHIN THE PAST 5 YEARS AT Q23b (e.g., FIRST 4 OPTIONS) ASK] For the next 3 questions please think about the LAST TIME your child experienced a severe allergic reaction while dining out or as a result of ordering food from a restaurant or foodservice establishment.

[IF ADULT & WITHIN THE PAST 5 YEARS AT Q23b (e.g., FIRST 4 OPTIONS) ASK] For the next 3 questions please think about the LAST TIME you experienced a severe allergic reaction while dining out or as a result of ordering food from a restaurant or foodservice establishment.

25a. Were you….

Dining out in a restaurant	❑
Ordering food directly from a restaurant (take-out or delivery)	❑
Ordering food through a third-party delivery service / app	❑

25b. **[PARENT]** Had the establishment been informed of your child's food allergy?

[ADULT] Had the establishment been informed of your food allergy?

Yes	❑
No	❑
Don't remember	❑

25c. Did the reaction require the use of an epinephrine auto-injector and / or trip to the hospital?

(Select all that apply)

Use of an epinephrine auto-injector	❑
Trip to hospital	❑
Neither use of an epinephrine auto-injector or trip to the hospital	❑
Don't remember	❑

26. **[IF RESPONDENT CHECKS "milk", "egg", "fish" or "shellfish" AT Q7 ASK]** When you see menu items listed or classified as **vegan**, which of the following types of allergies would you interpret these menus items as being safe for?

(Select ALL that apply)

[RANDOMIZE]

People with a milk or dairy allergy	❑
People with an egg allergy	❑
People with a fish allergy	❑
People with a shellfish allergy	❑
**[ALWAYS SHOW LAST]** None of the above	❑

[SHOW Q27a & Q27b ON SAME SCREEN – ASK TO PARENTS & ADULTS ONLY]

27a. **[ASK TO PARENTS & ADULTS]** When it comes to restaurants and foodservice establishments, who, if any restaurant, do you consider **"best-in-class"** when it comes to food allergy management and prevention?

(Enter name of restaurant / establishment below)

No restaurant is doing a good job ❑ **[IF RESPONDENT CHOOSES THIS OPTION GREY OUT / REMOVE OPEN-END BELOW]**

27b. **[ASK TO PARENTS & ADULTS]** Please explain why you consider this establishment to be best-in-class for food allergy management and prevention.

28. **[ASK TO PARENTS & ADULTS]** Some restaurants provide an allergy statement on their menu or website indicating that menu items may have come in contact with a food allergen(s) or that cross-contamination may occur. When you see this sort of messaging how do you interpret it? What does it mean to you?

29. **[ASK TO PARENTS & ADULTS]** Some restaurants have the ingredient list of menu items readily available for patrons upon request. If a restaurant wanted to provide menu ingredient transparency to patrons, would you expect the restaurant to…

Provide an ingredient list for ALL menu items	❑
Provide an ingredient list for menu options deemed "allergy-suitable" or "allergy-friendly"	❑

30a. **[ASK TO PARENTS & ADULTS]** Which of the following steps or actions, if any, would be **MOST influential** in giving you the assurance needed to dine out / order food comfortably?

(Select UP TO 3)

[RANDOMIZE]

- All restaurant staff undergo mandatory food allergy training ❑
- Restaurant has a separate and dedicated area to prepare and cook meals for customers with food allergies ❑
- Equipment and surfaces are wiped down and washed prior to preparing food for customers with allergies ❑
- Ingredient list is available for all menu items ❑
- Restaurant has up-to-date epinephrine auto-injectors available as part of their first aid kid ❑
- Restaurant manager / chef confirms with you that the items you ordered are safe based on your allergens ❑
- When ordering online (directly through the restaurant or through a third-party delivery service) you get an email / text confirmation after placing you order confirming that the restaurant can accommodate your food allergy and the items you have ordered are safe ❑
- The restaurant has a message on their menu or website indicating that they take allergies seriously and to please inform them of any allergies ❑
- Restaurant uses icons to indicate which items are safe for which allergies ❑
- Wait staff / cashier confirms with you that the items you ordered are safe based on your allergens ❑
- Restaurant flags dishes that may contain common allergens ❑
- **[ALWAYS SHOW 2nd LAST]** Other (please specify)________________ ❑
- **[ALWAYS SHOW LAST]** None of the above ❑

[SHOW Q30b & Q30c ON SAME SCREEN]

30b. **[ASK TO PARENTS & ADULTS]** If a restaurant were to implement the actions outlined in the previous question, how safe would you feel dining out / ordering food from this restaurant?

- Very safe ❑
- Somewhat safe ❑
- Not very safe ❑
- Not at all safe ❑

30c. **[ASK TO PARENTS & ADULTS]** If a restaurant were to implement the actions outlined in the previous question, how loyal would you be to that restaurant?

- Very loyal ❑
- Fairly loyal ❑
- Somewhat loyal ❑
- Not very loyal ❑
- Not at all loyal ❑

<u>SECTION 4: Basic Data</u>

Finally, a few questions about you for data classification purposes.

31. Do you identify as…

- Male ❒
- Female ❒
- Non-binary / gender non-conforming ❒
- Prefer not to answer ❒

32. Finally, which of the following categories best reflects your total annual household income before taxes?

<$25,000	❑
$25,000 to $49,999	❑
$50,000 to $74,999	❑
$75,000 to $99,999	❑
$100,000 to $149,999	❑
$150,000 to $199,999	❑
$200,000+	❑
Prefer not to answer	❑

33. **[ASK TO FAC DATABASE RESPONDENTS ONLY]** If you have any further comments or questions on foodservice you would like to share, please let us know in the comment box below.

(A response to this question is optional)

[MAKE RESPONDING TO THIS QUESTION OPTIONAL]

Thank you for completing this survey. We greatly appreciate your feedback.

FOR FAC DATABASE RESPONDENTS: Route to Food Allergy Canada website

FOR PANEL RESPONDENTS: Route to panel
